# *In Vivo* Imaging of Brain Ischemia Using an Oxygen-Dependent Degradative Fusion Protein Probe

**DOI:** 10.1371/journal.pone.0048051

**Published:** 2012-10-19

**Authors:** Youshi Fujita, Takahiro Kuchimaru, Tetsuya Kadonosono, Shotaro Tanaka, Yoshiki Hase, Hidekazu Tomimoto, Masahiro Hiraoka, Shinae Kizaka-Kondoh, Masafumi Ihara, Ryosuke Takahashi

**Affiliations:** 1 Department of Neurology, Graduate School of Medicine, Kyoto University, Sakyo-ku, Kyoto, Japan; 2 Department of Biomolecular Engineering, Tokyo Institute of Technology Graduate School of Bioscience and Biotechnology, Nagatsuta-cho, Midori-ku, Yokohama, Japan; 3 Department of Biochemistry, School of Medicine, Tokyo Women's Medical University, Tokyo, Japan; 4 Department of Neurology, Mie University Graduate School of Medicine, Mie, Japan; 5 Department of Radiation Oncology and Image-applied Therapy, Kyoto University Graduate School of Medicine, Shogoin, Sakyo-ku, Kyoto, Japan; 6 Department of Regenerative Medicine and Research, Institute of Biomedical Research and Innovation, Minatojima, Chuo-ku, Kobe, Hyogo, Japan; Biological Research Centre of the Hungarian Academy of Sciences, Hungary

## Abstract

Within the ischemic penumbra, blood flow is sufficiently reduced that it results in hypoxia severe enough to arrest physiological function. Nevertheless, it has been shown that cells present within this region can be rescued and resuscitated by restoring perfusion and through other protective therapies. Thus, the early detection of the ischemic penumbra can be exploited to improve outcomes after focal ischemia. Hypoxia-inducible factor (HIF)-1 is a transcription factor induced by a reduction in molecular oxygen levels. Although the role of HIF-1 in the ischemic penumbra remains unknown, there is a strong correlation between areas with HIF-1 activity and the ischemic penumbra. We recently developed a near-infrared fluorescently labeled-fusion protein, POH-N, with an oxygen-dependent degradation property identical to the alpha subunit of HIF-1. Here, we conduct *in vivo* imaging of HIF-active regions using POH-N in ischemic brains after transient focal cerebral ischemia induced using the intraluminal middle cerebral artery occlusion technique in mice. The results demonstrate that POH-N enables the *in vivo* monitoring and *ex vivo* detection of HIF-1-active regions after ischemic brain injury and suggest its potential in imaging and drug delivery to HIF-1-active areas in ischemic brains.

## Introduction

Hypoxia-inducible factor 1 (HIF-1) is activated by a variety of stimuli, including focal cerebral ischemia [1]. HIF-1 is a heterodimeric transcription factor consisting of an oxygen-regulated alpha subunit (HIF-1α) and a constitutively expressed beta subunit (HIF-1ß), which play a central role in cellular adaptation by regulating a wide array of genes in response to limited oxygen availability [2]. Under normoxia, prolyl hydroxylases (PHDs) hydroxylate specific proline residues of the oxygen-dependent degradation domain (ODD) of HIF-1α, leading to its polyubiquitination by the von Hippel–Lindau protein (VHL) and subsequent proteasomal degradation. In contrast, hypoxia abrogates prolyl hydroxylation by PHDs and, after VHL binding to HIF-1α, leads to the stabilization and accumulation of HIF-1α [3], [4], [5].

Oxygenation of brain tissue is impaired as a result of occlusion of a cerebral blood vessel causing subsequent irreversible infarction. The infarct core is surrounded by a hypoxic area [6], known as the ischemic penumbra [7], a region of hypoperfused, functionally impaired, but still viable tissue, in which HIF-1 activation is observed [8], [9]. Therefore, the HIF-1-active region in the ischemic brain provides a suitable target for efficiently treating cerebral infarction.

**Figure 1:**
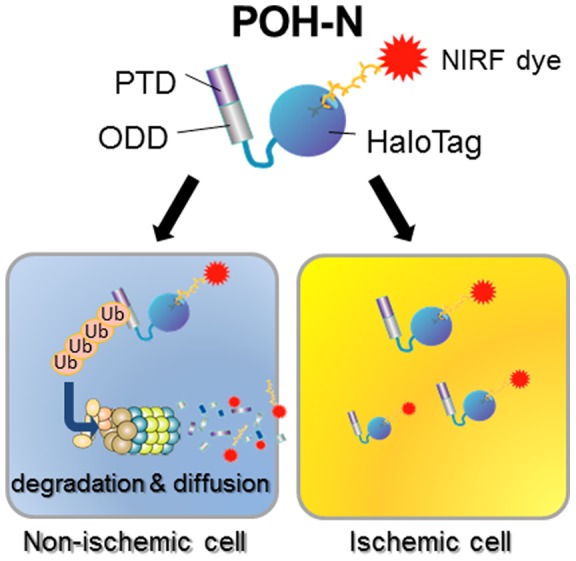
POH-N probe structure. Under normoxic conditions, POH-N is immediately degraded via VHL-mediated ODD, and the resultant POH-N fragments diffuse from the cells. In contrast, POH-N is more stable in HIF-1-active cells, thus creating a contrast between HIF-1-active and HIF-1-inactive cells.

We previously reported that a fusion protein containing the ODD_548–603_ of human HIF-1α is efficiently degraded under normoxic conditions, via a VHL-mediated protein degradation system, in a manner similar to that of HIF-1α [10]. Using ODD-dependent degradation as a target-specific distribution and taking advantage of the capability of the protein-transduction domain (PTD) fusion protein to penetrate the cell membrane, we have developed PTD-ODD fusion proteins that specifically target HIF-1-active cancer cells *in vivo* [11]–[15]. We recently created a near-infrared fluorescent (NIRF)-labeled PTD-ODD-HaloTag (POH) that functions as an imaging probe specific to HIF-1-active cancer cells both *in vitro* and *in vivo* [16] (Fig. 1). Because NIRF-labeled POH (POH-N) is efficiently delivered to regions with less blood flow [11] and the PTD fusion protein can penetrate the blood–brain barrier [17], POH may be applicable to ischemic brain diseases as a specific probe for detecting HIF-1-active ischemic penumbra.

Here, we investigated the performance of POH-N as a molecular probe for imaging and targeting HIF-1-active regions in an ischemic stroke mouse model. The results demonstrate that POH-N allows *in vivo* monitoring and *ex vivo* detection of the HIF-1-active regions after ischemic brain injury.

## Materials and Methods

### Ethics statement

All animal experiments in this study were performed with the approval of the Animal Experiment Committees of Kyoto University, Graduate School of Medicine (Permit Number: MedKyo10202) and in strict accordance with the relevant national and international guidelines.

### Animal preparation

**Figure 2:**
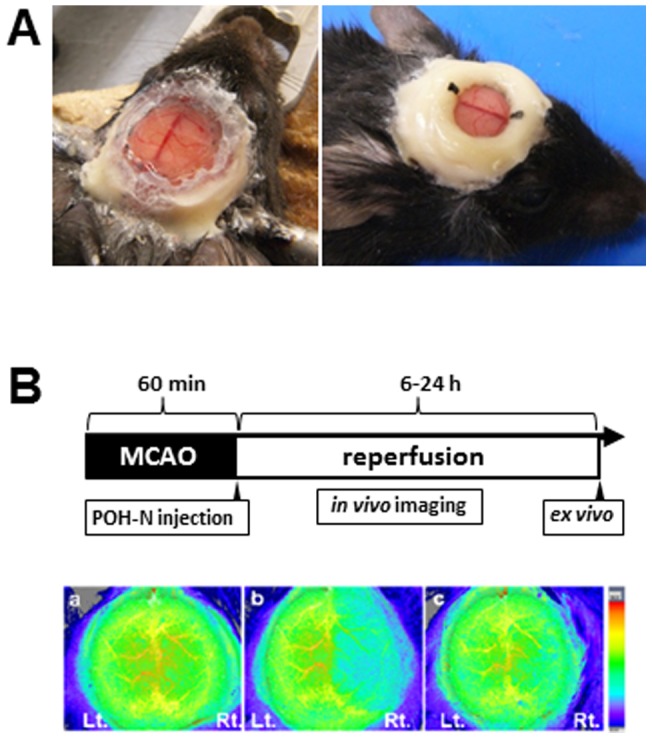
Experimental design. (A) Cranial window in a C57/BL6 mouse. Experimental design of the closed cranial window. (B) Experimental design (upper panel). Representative two-dimensional images of cerebral blood flow measured by laser speckle perfusion imaging before MCAO (a), during MCAO (b), and after reperfusion (c) are shown in the lower panels. MCAO: middle cerebral artery occlusion.

The cranial window surgical procedure was performed for *in vivo* imaging, as previously described [18]. In brief, male C57BL/6J mice (6–7 weeks old) were anesthetized with 1.5% isoflurane in air, via a snout mask. A 6-mm-diameter hole was made using a fine drill bit in the skull. The center of the cranial window was located 2 mm posterior to the bregma on the midline. The dura mater was left intact. To cover the hole, an 8-mm cover glass (0.45–0.60-mm-thick) was sealed to the skull with histocompatible cyanoacrylate glue and dental cement, which adhered to the bone (Fig. 2A). Transient focal cerebral ischemia was induced using the intraluminal middle cerebral artery (MCA) occlusion (MCAO) technique [19]. Body temperature was maintained at 37°C using a feedback-controlled heating pad. An incision was made into the external carotid artery, and a silicon-coated 8–0 nylon monofilament was inserted through the right internal carotid artery to occlude the MCA at its origin. After 60 min of occlusion, blood flow was restored by withdrawing the nylon suture. For generation of permanent occlusion of MCA, the nylon suture was not withdrawn. The survival rate of the MCAO/R model and the permanent MCAO model was more than 90% 24 hours after operation. Animals were assessed using laser speckle perfusion imaging (Omegazone; Omegawave Inc., Tokyo, Japan) to confirm adequate induction of focal ischemia and successful reperfusion (Fig. 2B).

### Plasmid construction and preparation of fusion proteins

The plasmid encoding the POH protein was constructed by substituting the coding sequences of procaspase-3 in PTD-ODD-procaspase-3 with HaloTag (Promega, Madison, WI), as previously described [16]. The plasmid encoding POmH containing the point substitution mutation, P564G [15], was prepared using a QuickChange XL site-directed mutagenesis kit (Stratagene, La Jolla, CA) at the proline residue corresponding to HIF-1α P564. Final cDNA constructs were inserted into the pGEX-6P-3 plasmids (GE Healthcare Bio-Science Corp., Piscataway, NJ). Fusion proteins were expressed in BL21-CodonPlus cells (Stratagene, La Jolla, CA) as GST-tagged proteins. These GST-tagged proteins were purified with a GST column and digested with precision protease (GE healthcare Bio-Science Corp., Piscataway, NJ) to remove GST tags from the fusion proteins. The final products were equilibrated in Mg^2+^/Ca^2+^ free PBS (pH 8.0).

**Figure 3:**
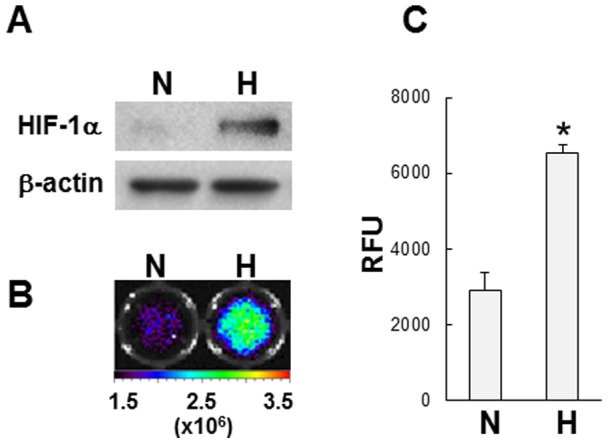
Stabilization of POH-N under hypoxic conditions. SH-SY5Y neuroblastoma cells cultured under normoxic (N) or hypoxic (H) conditions were treated with POH probe. (A) HIF-1α protein levels were analyzed by western blotting (a representative blot is shown). (B) The fluorescence intensity of POH probe in cells was measured. (C) Representative fluorescence images are shown. *P<0.02 (vs. normoxic condition).

**Figure 4:**
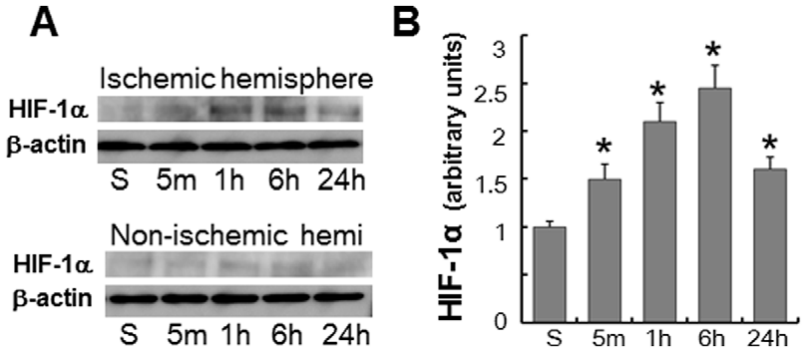
HIF-1α accumulation after focal brain ischemia. (A) Western blot analysis of HIF-1α in the ischemic and non-ischemic hemispheres of mice subjected to MCAO followed by reperfusion. (B) Densitometric analysis of HIF-1α protein levels in the ischemic hemispheres. Data were normalized relative to β-actin levels, and the values obtained from sham-operated controls (S) were arbitrarily defined as 1. *P<0.05 (vs. sham, n = 4).

### Preparation of the POH-N probe

We used NIRF dye IR800, as previously described [16]. The HaloTag ligand-IR800 was provided by Promega Corporation. HaloTag ligands (1 mg, 2.87 μmol; Promega, Madison, WI) in 100 µL of dimethyl formamide (DMF) were mixed with the NIRF dye and succinimidyl ester (1 mg, ∼0.8 µmol; Invitrogen, Carlsbad, CA) in 1 mL of 10 mM boric acid (pH 8.5) in DMF. The reaction mixture was stirred in the dark for 12 h at room temperature. The reaction mixture was applied to a SepPak C18 reverse-phase column (Waters, Milford, MA), and the HaloTag ligands labeled with NIRF dye (HL-N) were resolved in 100 µL DMF. POH protein (40 µmol/L) was mixed with three volumes of HL-N (120 nmol/15 µL) in 10 mL PBS (pH 8.0) containing 100 mM Tris-HCl (pH 8.0) and 3 M (NH_4_)_2_SO_4_ for 2 h. POH-N probes were subsequently purified with a PD-10 gel filtration column (GE healthcare Bio-Science Corp., Piscataway, NJ) and an Amicon-10 centrifugation column (Millipore, Billerica, MA). Purified POH-N was finally resolved in PBS (pH 8.0). Fluorescence characterizations were confirmed by SDS-PAGE fluorescence imaging. The labeling rate, calculated as described by the manufacturer, was >0.7 [16].

**Figure 5:**
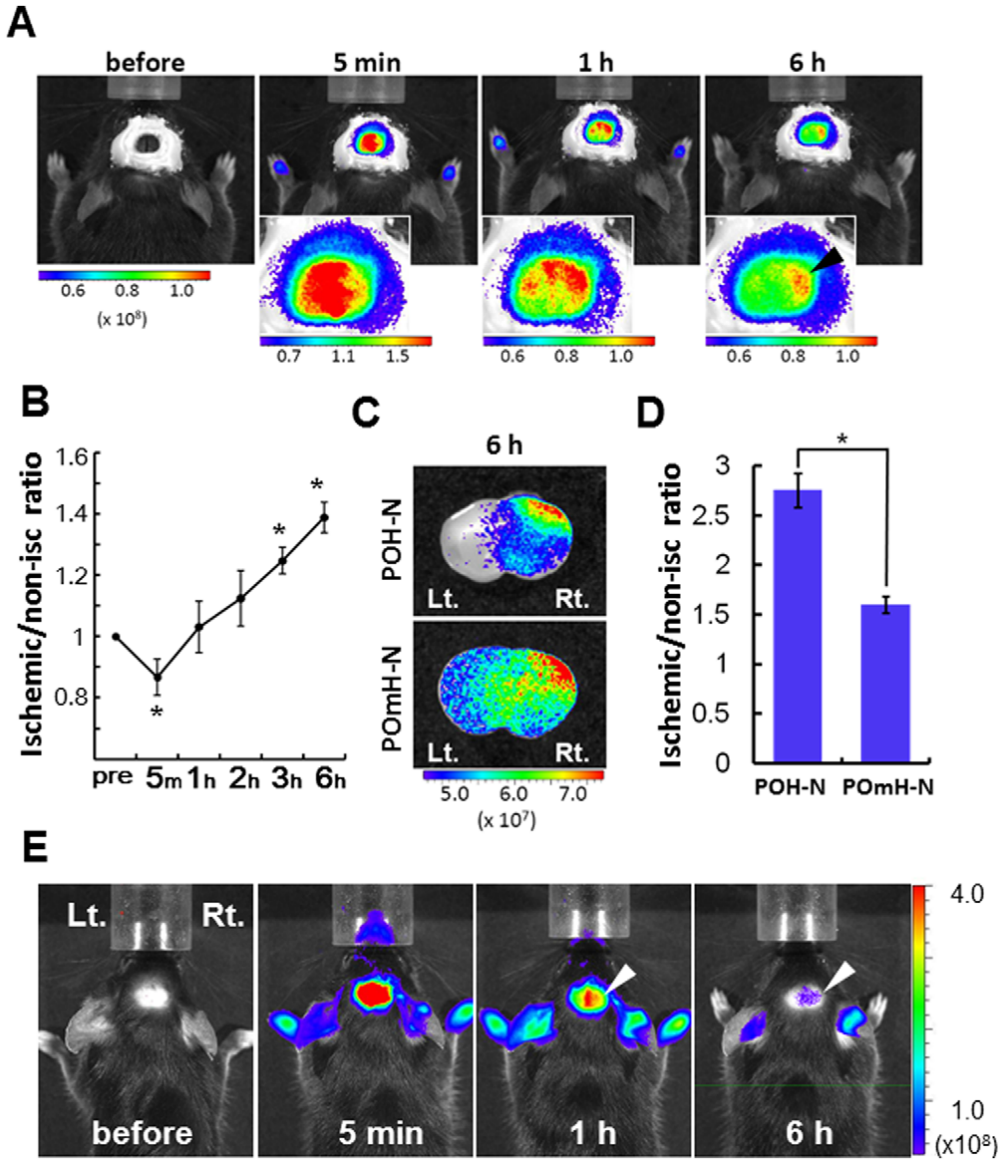
Imaging of HIF-1-active regions in the focal brain ischemia model. (A) Representative *in vivo* fluorescence images visualized through a cranial window before and at 5 min, 1 h, and 6 h after POH-N administration are shown. Magnified head images are shown in the lower left panels. Arrowheads indicate accumulation of the probe in the right ischemic hemisphere. (B) The relative fluorescence intensity of the ischemic hemisphere to the non-ischemic hemisphere. Fluorescence intensities were measured at the indicated times after POH-N administration. *P<0.05, n = 3. (C) *Ex vivo* imaging of the coronal brain sections after POH-N injection. (D) Relative fluorescence of the ischemic hemisphere compared with the non-ischemic hemisphere at 6 h after probe administration (n = 3/group: *P<0.05). Relative fluorescence values were calculated using ROIs mirrored along the midline of the cerebral hemispheres. (E) *In vivo* fluorescence images visualized without preparation of a cranial window before and at 5 min, 1 h, and 6 h after POH-N administration. Anesthetized C57BL/6J mice were shaved and depilated top of the head 24 h before experimentation. Arrowheads indicate accumulation of the probe in the right ischemic hemisphere.

### *In vitro* fluorescence measurement

SH-SY5Y neuroblastoma cells (2×10^5^ cells/well) were seeded in a six-well plate (Riken Cell Bank, Tsukuba, Japan). The cells were pre-incubated under hypoxic (1% O_2_) or normoxic (21% O_2_) conditions for 16 h. The probe (500 nM) was then added, followed by incubation for 1 h. The cells were then washed with fresh medium, incubated for 3 h in fresh medium, and suspended in 200 μL of radioimmunoprecipitation assay (RIPA) buffer. Fluorescence was measured and imaged for 150-μL aliquots of suspension in a 96-well plate using an Infinite® F500 microplate reader (Tecan, Durham, NC) with excitation and emission filters at 740±25 and 780±20 nm, and the IVIS®-Spectrum *in vivo* imaging system (Caliper Life Sciences, Alameda, CA) with excitation and emission filters at 710±15 and 800±10 nm, respectively.

**Figure 6:**
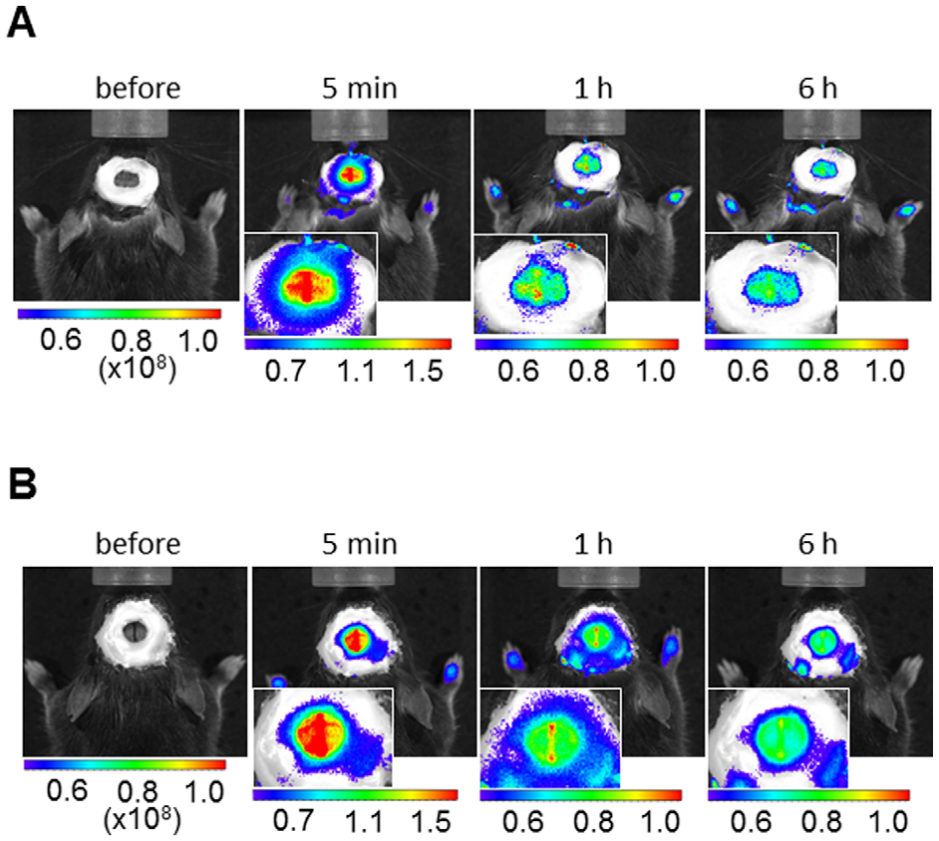
No clear visualization of HIF-1-active regions in the permanent brain ischemia model or with delayed injection of POH-N in the focal brain ischemia model. (A) Representative *in vivo* fluorescence images visualized through a cranial window before and at 5 min, 1 h, and 6 h after POH-N administration are shown. POH-N was injected intravenously at 60 min after permanent MCA occlusion. (B) Representative *in vivo* fluorescence images visualized through a cranial window before and at 5 min, 1 h, and 6 h following POH-N administration at 24 h after reperfusion. Magnified head images are shown in the lower left panels.

### Western blot analysis

To analyze cultured cells, SH-SY5Y cells were seeded in a six-well plate. The cells were pre-incubated under hypoxic or normoxic conditions for 6 h, then washed with medium, incubated for 3 h, and lysed using 200 μL of Laemmli sample buffer. Brain tissue samples were homogenized with a Dounce glass homogenizer using ice-cold RIPA buffer supplemented with protease inhibitors (Nacalai Tesque, Kyoto, Japan). Lysates were centrifuged at 10,000× *g* for 10 min at 4°C, and supernatants were collected. Protein concentrations were determined by the BCA protein assay (Pierce, Rockford, IL). Protein samples were electrophoresed on 10% SDS-polyacrylamide gel and transferred to PVDF membranes. The POH-N probes, β-actin and HIF-1α, were detected by a monoclonal anti-β-actin antibody (Sigma-Aldrich, St. Louis, MO) and a polyclonal anti-HIF-1α antibody (R&D Systems, Minneapolis, MN), respectively. The primary antibodies were then reacted with appropriate secondary horseradish peroxidase-conjugated antibodies (GE Healthcare Bio-Science Corp., Piscataway, NJ). Signals were detected using the chemiluminescence ECL-PLUS system (GE Healthcare Bio-Science Corp., Piscataway, NJ). Data were normalized relative to the β-actin levels and expressed as percentages of the sham-operated controls.

**Figure 7:**
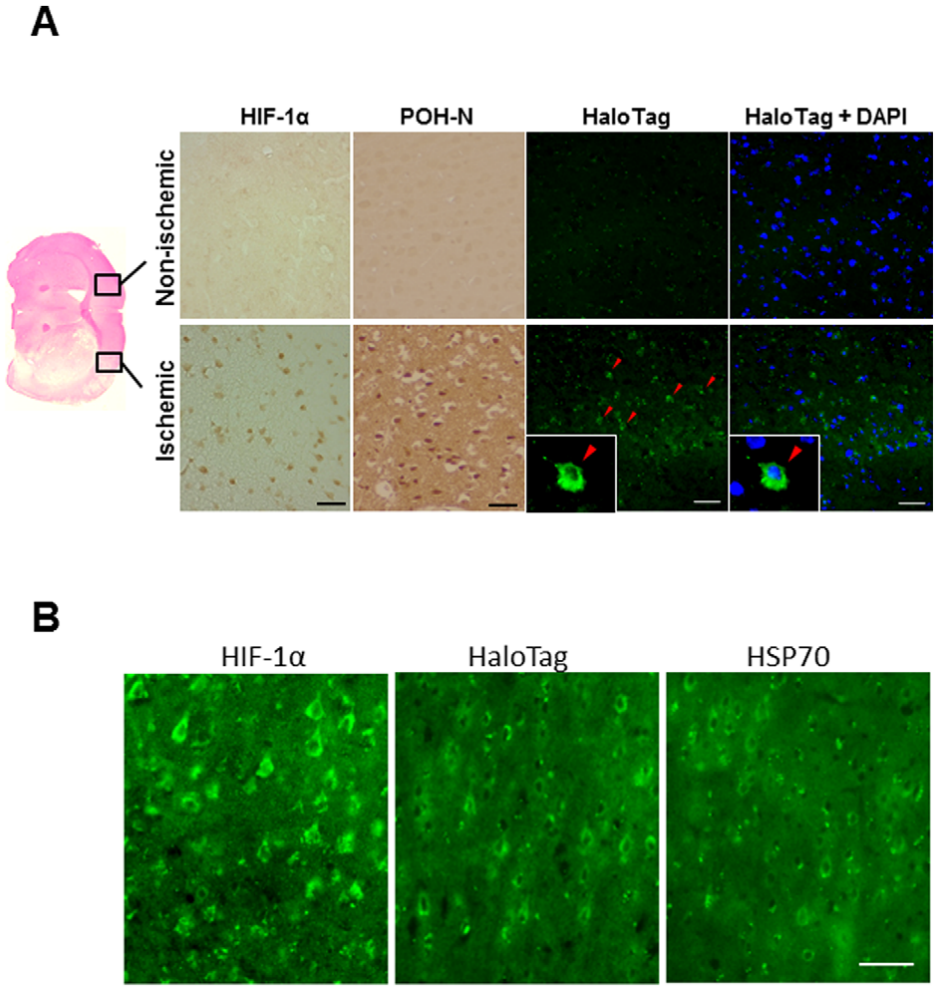
Immunohistochemical detection of HIF-1-active cells and POH-N probe. (A) Immunohistochemical analysis of HIF-1α, POH-N (ODD) and HaloTag (green), with or without DAPI nuclear staining (blue), at 1 day after probe administration. Panels at the bottom show magnified images. (B) Similar distributions of HIF-1α, HaloTag, and HSP70 in pyramidal neurons of the cortical layer bordering the infarct. Scale bars, 50 μm.

### *In vivo* and *ex vivo* fluorescence imaging

POH probe (2 nmol) in 100 μL PBS (pH 8.0) was injected intravenously into the tail vein at 5 min, 6 hours, and 24 hours after reperfusion by the withdrawal of the nylon suture. Alternatively, POH probe (2 nmol) was injected intravenously at 60 min after permanent MCAO, without withdrawal of the nylon suture. Fluorescence images were acquired at the indicated times after injections. All fluorescence images were acquired with the IVIS®-Spectrum (Caliper Life Sciences, Alameda, CA) system, using the following parameters: excitation filter, 710±15 nm; emission filter, 800±10 nm; exposure time, 1 s; binning, small; field of view, 6×6 cm; and f-stop, 1. Some mice were sacrificed after *in vivo* imaging, and their brains were harvested and sliced into 3-mm-thick coronal sections. Fluorescence emissions from these brain sections were measured using the IVIS®-Spectrum system, under the same set of parameters for the excitation filter, emission filter, and exposure time. Relative fluorescence values were calculated by using regions of interest (ROIs) mirrored along the midline of the cerebral hemispheres. The contribution of the ODD domain in POH to clearance acceleration in the non-ischemic brains was examined using POmH-N, which has a point mutation corresponding to human HIF-1α (P564G) in the ODD domain and thus lacks ODD regulation [20].

### Immunohistochemical analyses

Brain cryosections (10-μm-thick) were prepared using a cryostat (Leica CM3050S; Leica Microsystems, Wetzlar, Germany) and fixed in 4% paraformaldehyde. Cryosections were immunolabeled with the following primary antibodies: rabbit polyclonal anti-HIF-1α antibody (R&D Systems, Minneapolis, MN), rabbit polyclonal anti-ODD antibody [16], rabbit polyclonal anti-HaloTag antibody (Promega, Madison, WI), and rabbit polyclonal anti-HSP70 antibody (Cell Signaling Technology, Danvers, MA). Primary antibodies were applied overnight at 4°C. The sections were then incubated with biotin- or FITC-conjugated secondary antibodies. The avidin–biotin–peroxidase complex (ABC) (ABC-Elite; Vector Laboratories, Burlingame, CA) was applied, and the reaction product was visualized using diaminobenzidine (DAB). All photos were taken using a BZ-9000 microscope (Keyence, Osaka, Japan).

### Statistical analysis

Data are presented as mean ± SEM. Statistical analyses were performed using ANOVA. Values of *P*<0.05 were considered statistically significant.

## Results

### Stabilization of POH-N under hypoxic conditions *in vitro*

HIF-1α protein levels increased under hypoxic conditions compared with normoxic conditions (Fig. 3A). When SH-SY5 neuroblastoma cells were treated with POH-N, significantly (*P<0.02) higher fluorescent signals were detected in cells cultured in hypoxic conditions compared with normoxic conditions in a manner similar to that of HIF-1α protein levels (Fig. 3B and C).

### HIF-1α accumulation after focal cerebral ischemia

Quantitative western blot analysis showed that cerebral ischemia induced by transient MCAO triggered a significant (*P<0.05, n = 4) increase in the HIF-1α protein levels in the ischemic hemisphere (Fig. 4A and B). HIF-1α protein levels reached a peak at 6 h after 60 min MCAO in the ischemic hemisphere and declined thereafter (Fig. 4A and B).

### *In vivo* imaging of HIF-1-active regions in an ischemic stroke model

To examine the possible application of POH-N to ischemic diseases, we administered POH-N in mice with focal cerebral ischemia induced by transient MCAO. The fluorescent signal for POH-N was measured at the indicated times (Fig. 5A and B). Five minutes after POH-N administration, fluorescent signals were lower in the ischemic (right) hemisphere than in the non-ischemic (left) hemisphere, probably reflecting post-ischemic hypoperfusion in the ischemic hemisphere. However, at 1–6 h after POH-N administration, the fluorescence intensity increased in the ischemic hemisphere and decreased in the non-ischemic hemisphere. At 3–6 hours after POH-N administration, the relative fluorescence intensity of the ischemic hemisphere was significantly greater than the baseline, compared to that of the non-ischemic hemisphere (ischemic/non-ischemic ratio) (Fig. 5B).

### ODD-dependent clearance acceleration in the non-ischemic brains

Examination of the coronal brain sections confirmed that the fluorescent signal was derived from the ischemic hemisphere, particularly in the cortical region adjacent to the striatum (infarct core), at 6 h after POH-N administration (Fig. 5C). Although the ischemic sites showed higher fluorescence intensity than the non-ischemic sites in both POH-N- and POmH-N-injected brains, the fluorescent signals in POH-N-injected brains were more restricted to the ischemic region (Fig. 5C). Furthermore, the non-ischemic sites in POH-N-injected brains showed significantly lower relative fluorescence intensities than those in POmH-N-injected brains (Fig. 5C and D). The fluorescent signal derived from the ischemic hemisphere was visualized even without cranial window (Fig. 5E). However, when POH-N was injected intravenously at 60 min after permanent MCAO or at 24 hours after reperfusion in the transient MCAO, fluorescence intensity was not different between the ischemic and non-ischemic hemispheres (Fig. 6A and B).

### Specificity of POH-N to HIF-1α-positive cells in the ischemic brain

Occlusion of the MCA for 60 min induced reproducible ischemic infarcts in the striatum (infarct core) and cerebral cortex, as detected by histology. The specific localization of POH probe in HIF-1-active cells was examined by immunohistochemical analysis of the brain at 24 h after POH-N injection. POH protein was specifically detected in the ischemic cerebral cortex, where abundant HIF-1α-positive cells were also observed (Fig. 7). POH protein was mainly localized to the cytoplasm of cells (magnified images in Fig. 5 bottom panels), which is concordant with a previous report [16]. HIF-1α, HaloTag, and HSP70 showed similar expression patterns in cortical pyramidal neurons within the ischemic penumbra. Overall, these results demonstrate the specificity of POH to HIF-1-active ischemic, but potentially salvageable, cells.

## Discussion

*In vivo* imaging using the POH probe was previously demonstrated to accurately identify HIF-1-active regions in a mouse cancer model [16]. The present study shows that POH-N can also detect HIF-1-active ischemic lesions in a mouse focal cerebral ischemia model. One-hour focal ischemia induced HIF-1 upregulation at 1, 6, and 24 hours post-ischemia, even after reperfusion, probably reflecting the ‘no-reflow’ phenomenon [21]. Although the tissue in the ischemic hemisphere may have been temporarily subject to relative hyperoxic status after reperfusion, the fluorescent POH system worked at least 24 hours post ischemia, thus enabling HIF-1 imaging. This POH fusion protein method therefore has great potential in improving the diagnosis and treatment of ischemic stroke.

The PTD-mediated delivery system has been demonstrated to enable the delivery of biologically active proteins across the blood–brain barrier. It has been shown that fusion proteins containing the PTD sequence, derived from HIV trans-activator of transcription (TAT), are delivered into the brain tissue after systemic administration [17]. To date, the efficacy of PTD fusion proteins, including the anti-apoptotic protein Bcl-xL, neurotrophic factor GDNF, and antioxidant enzyme SOD, have been demonstrated in rodent models of cerebral ischemia [22], [23], [24], [25]. However, many neuroprotective drugs that have shown promise in experimental animal models have failed to achieve positive results during clinical trials [26], [27]. One of the reasons for such failures is that the target drug levels identified in animals cannot be tolerated by stroke patients. For example, an N-methyl-D-aspartate (NMDA) receptor antagonist has been shown to protect against ischemic stroke at plasma levels greater than 40 μg/mL in animal models; however, the highest tolerable dose in stroke patients is only half of this target level, above which neurological and psychiatric adverse effects are observed [27], [28]. One potential way to circumvent such adverse effects would be to take advantage of the ODD-mediated acceleration of clearance under normoxic conditions. In our experiments, POH-N was cleared from the non-ischemic hemisphere significantly faster than POmH-N (Fig. 5). The results strongly support previous reports stating that the ODD domain contributes to the rapid clearance of POH-N from normoxic HIF-inactive tissue [16].

The ischemic penumbra, which is the functionally impaired but potentially viable tissue surrounding the infarct core, is currently considered to be the most promising target for ischemic stroke therapy. However, the accurate identification of patients exhibiting penumbral damage is not straightforward. Currently, the most widely accepted and practical method for identifying the ischemic penumbra in stroke patients is to look for an ischemic region displaying reduced perfusion on MRI but a normal signal on diffusion-weighted imaging [29]. However, several studies show that this interpretation of diffusion- and perfusion-weighted imaging may be an oversimplification [30]. Although the ischemic penumbra was originally defined on the basis of cerebral blood flow and physiological parameters [7], it can also be described in molecular terms [8] by examination of molecular layers emanating from the infarct core. Specifically, pro-apoptotic proteins and anti-apoptotic heat shock protein 70 are expressed in the layer bordering the infarct, and HIF in the layers beyond [31], [32], [33]. Furthermore, ischemia-induced spreading depression induces the expression of c-fos and many other immediate early genes in the outer layer [34], although such identification methods have not yet been applied in humans.

In the present study, POH-N was delivered to ischemic lesions, including peri-infarct regions (Fig. 7). This result supports the idea that the PTD allows fusion proteins to be delivered to hypoperfused tissue, most likely via diffusion, to achieve the molecular definition of an ischemic penumbra. Furthermore, POH-N significantly accumulated in the ischemic regions and was specifically detected in HIF-1-active cortical cells after focal brain ischemia (Fig. 7). We concede that this imaging technique may be deemed inferior, in terms of resolution, when compared to more established imaging techniques, such as MRI. However, such fluorescent imaging techniques may provide a useful complement to existing imaging techniques, as bedside evaluation would be available without the need of transferring stroke patients to the diagnostic radiology unit. In addition, since HaloTag ligands can be conjugated to a wide range of biomaterials. POH offers a wide range of clinical applications, including the production of imaging probes, even for MRI. Furthermore, a POH-mediated delivery system could be used to selectively target drugs to the ischemic penumbra, an area that has potential for recovery and thus may provide a target for medical interventions.

A limitation that became apparent during this study was the lack of clarity of some images of small mouse brains captured with the IVIS®-Spectrum, which hindered the clear discrimination between ischemic core and penumbra. Another limitation arose through POH-N failing to reach ischemic lesions in the permanent MCAO model. In addition, POH-N had to be injected immediately, not at 6 or 24 hours, after reperfusion to visualize HIF-1-active regions even in the transient MCAO model. Since the partial or complete recanalization rate of major vessel occlusion exceeds 50% in the tPA era [35], clinical application of this *in vivo* fluorescence imaging system should be further explored in parallel with efforts to enhance imaging sensitivity and widen the narrow time window.

## References

[1] Bergeron M, Yu AY, Solway KE, Semenza GL, Sharp FR (1999) Induction of hypoxia-inducible factor-1 (HIF-1) and its target genes following focal ischemia in rat brain. Eur J Neurosci 11: 4159–4170. 10594641 10.1046/j.1460-9568.1999.00845.x

[2] Semenza GL (2000) HIF-1: mediator of physiological and pathophysiological responses to hypoxia. J Appl Physiol 88: 1474–1480. 10749844 10.1152/jappl.2000.88.4.1474

[3] Kaelin WG (2005) Proline hydroxylation and gene expression. Annu Rev Biochem 74: 115–128. 15952883 10.1146/annurev.biochem.74.082803.133142

[4] Schofield CJ, Ratcliffe PJ (2004) Oxygen sensing by HIF hydroxylases. Nat Rev Mol Cell Biol 5: 343–354. 15122348 10.1038/nrm1366

[5] Tanimoto K, Makino Y, Pereira T, Poellinger L (2000) Mechanism of regulation of the hypoxia-inducible factor-1 alpha by the von Hippel–Lindau tumor suppressor protein. EMBO J 19: 4298–4309. 10944113 10.1093/emboj/19.16.4298 PMC302039

[6] Marti HJ, Bernaudin M, Bellail A, Schoch H, Euler M, et al (2000) Hypoxia-induced vascular endothelial growth factor expression precedes neovascularization after cerebral ischemia. Am J Pathol 156: 965–976. 10702412 10.1016/S0002-9440(10)64964-4 PMC1876841

[7] Astrup J, Siesjo BK, Symon L (1981) Thresholds in cerebral ischemia—the ischemic penumbra. Stroke 12: 723–725. 6272455 10.1161/01.str.12.6.723

[8] Sharp FR, Lu A, Tang Y, Millhorn DE (2000) Multiple molecular penumbras after focal cerebral ischemia. J Cereb Blood Flow Metab 20: 1011–1032. 10908035 10.1097/00004647-200007000-00001

[9] Bergeron M, Yu AY, Solway KE, Semenza GL, Sharp FR (1999) Induction of hypoxia-inducible factor-1 (HIF-1) and its target genes following focal ischaemia in rat brain. Eur J Neurosci 11: 4159–4170. 10594641 10.1046/j.1460-9568.1999.00845.x

[10] Harada H, Kizaka-Kondoh S, Hiraoka M (2006) Mechanism of hypoxia-specific cytotoxicity of procaspase-3 fused with a VHL-mediated protein destruction motif of HIF-1alpha containing Pro564. FEBS Lett 580: 5718–5722. 17010341 10.1016/j.febslet.2006.09.025

[11] Harada H, Hiraoka M, Kizaka-Kondoh S (2002) Antitumor effect of TAT-oxygen-dependent degradation-caspase-3 fusion protein specifically stabilized and activated in hypoxic tumor cells. Cancer Res 62: 2013–2018. 11929818

[12] Harada H, Kizaka-Kondoh S, Hiraoka M (2005) Optical imaging of tumor hypoxia and evaluation of efficacy of a hypoxia-targeting drug in living animals. Mol Imaging 4: 182–193. 16194450 10.1162/15353500200505112

[13] Harada H, Kizaka-Kondoh S, Li G, Itasaka S, Shibuya K, et al (2007) Significance of HIF-1-active cells in angiogenesis and radioresistance. Oncogene 26: 7508–7516. 17563752 10.1038/sj.onc.1210556

[14] Hiraga T, Kizaka-Kondoh S, Hirota K, Hiraoka M, Yoneda T (2007) Hypoxia and hypoxia-inducible factor-1 expression enhance osteolytic bone metastases of breast cancer. Cancer Res 67: 4157–4163. 17483326 10.1158/0008-5472.CAN-06-2355

[15] Kizaka-Kondoh S, Itasaka S, Zeng L, Tanaka S, Zhao T, et al (2009) Selective killing of hypoxia-inducible factor-1-active cells improves survival in a mouse model of invasive and metastatic pancreatic cancer. Clin Cancer Res 15: 3433–3441. 19417024 10.1158/1078-0432.CCR-08-2267

[16] Kuchimaru T, Kadonosono T, Tanaka S, Ushiki T, Hiraoka M, et al (2010) In vivo imaging of HIF-active tumors by an oxygen-dependent degradation protein probe with an interchangeable labeling system. PLoS ONE 5: e15736. 21203417 10.1371/journal.pone.0015736 PMC3009742

[17] Schwarze SR, Ho A, Vocero-Akbani A, Dowdy SF (1999) In vivo protein transduction: delivery of a biologically active protein into the mouse. Science 285: 1569–1572. 10477521 10.1126/science.285.5433.1569

[18] Yuan F, Salehi HA, Boucher Y, Vasthare US, Tuma RF, et al (1994) Vascular permeability and microcirculation of gliomas and mammary carcinomas transplanted in rat and mouse cranial windows. Cancer Res 54: 4564–4568. 8062241

[19] Shah ZA, Namiranian K, Klaus J, Kibler K, Doré S (2006) Use of an optimized transient occlusion of the middle cerebral artery protocol for the mouse stroke model. J Stroke Cerebrovasc Dis 15: 133–138. 17904065 10.1016/j.jstrokecerebrovasdis.2006.04.002

[20] Chan DA, Sutphin PD, Yen SE, Giaccia AJ (2005) Coordinate regulation of the oxygen-dependent degradation domains of hypoxia-inducible factor 1 alpha. Mol Cell Biol 25: 6415–6426. 16024780 10.1128/MCB.25.15.6415-6426.2005 PMC1190339

[21] Hase Y, Okamoto Y, Fujita Y, Kitamura A, Ito H, Maki T, et al (2012) Cilostazol, a phosphodiesterase inhibitor, prevents no-reflow and hemorrhage in mice with focal cerebral ischemia. Exp Neurol 233: 523–533. 22173318 10.1016/j.expneurol.2011.11.038

[22] Cao G, Pei W, Ge H, Liang Q, Luo Y, et al (2002) In vivo delivery of a Bcl-xL fusion protein containing the TAT protein transduction domain protects against ischemic brain injury and neuronal apoptosis. J Neurosci 22: 5423–5431. 12097494 10.1523/JNEUROSCI.22-13-05423.2002 PMC6758230

[23] Kilic E, Dietz GP, Hermann DM, Bahr M (2002) Intravenous TAT–Bcl-Xl is protective after middle cerebral artery occlusion in mice. Ann Neurol 52: 617–622. 12402259 10.1002/ana.10356

[24] Kilic U, Kilic E, Dietz GP, Bahr M (2003) Intravenous TAT–GDNF is protective after focal cerebral ischemia in mice. Stroke 34: 1304–1310. 12677018 10.1161/01.STR.0000066869.45310.50

[25] Kim DW, Eum WS, Jang SH, Kim SY, Choi HS, et al (2005) Transduced TAT–SOD fusion protein protects against ischemic brain injury. Mol Cells 19: 88–96. 15750345

[26] Fisher M, Bastan B (2008) Treating acute ischemic stroke. Curr Opin Drug Discov Devel 11: 626–632. 18729014

[27] Savitz SI, Fisher M (2007) Future of neuroprotection for acute stroke: in the aftermath of the SAINT trials. Ann Neurol 61: 396–402. 17420989 10.1002/ana.21127

[28] Labiche LA, Grotta JC (2004) Clinical trials for cytoprotection in stroke. NeuroRx 1: 46–70. 15717007 10.1602/neurorx.1.1.46 PMC534912

[29] Schlaug G, Benfield A, Baird AE, Siewert B, Lövblad KO, et al (1999) The ischemic penumbra: operationally defined by diffusion perfusion MRI. Neurology 53: 1528–1537. 10534263 10.1212/wnl.53.7.1528

[30] Kucinski T, Naumann D, Knab R, Schoder V, Wegener S, et al (2005) Tissue at risk is overestimated in perfusion-weighted imaging: MR imaging in acute stroke patients without vessel recanalization. Am J Neuroradiol 26: 815–819. 15814926 PMC7977137

[31] Nedergaard M (1987) Neuronal injury in the infarct border: a neuropathological study in the rat. Acta Neuropathol 73: 267–274. 3618118 10.1007/BF00686621

[32] Kinouchi H, Sharp FR, Koistinaho J, Hicks K, Kamii H, et al (1993) Induction of heat shock HSP70 mRNA and HSP70 kDa protein in neurons in the ‘penumbra’ following focal cerebral ischemia in the rat. Brain Res 619: 334–338. 8374789 10.1016/0006-8993(93)91630-b

[33] Wang GL, Semenza GL (1995) Purification and characterization of hypoxia-inducible factor 1. J Biol Chem 270: 1230–1237. 7836384 10.1074/jbc.270.3.1230

[34] Koistinaho J, Pasonen S, Yrjanheikki J, Chan PH (1999) Spreading depression-induced gene expression is regulated by plasma glucose. Stroke 30: 114–119. 9880398 10.1161/01.str.30.1.114

[35] Gonzalez RG (2006) Imaging-guided acute ischemic stroke therapy: From “time Is brain” to “physiology is brain”. Am J Neuroradiol 27: 728–735. 16611754 PMC8133997

